# ChatGPT and Corporations of Mega-journals Jeopardize the Norms That Underpin Academic Publishing

**DOI:** 10.34172/aim.2024.17

**Published:** 2024-02-01

**Authors:** Farid Rahimi, Amin Talebi Bezmin Abadi

**Affiliations:** ^1^Research School of Biology, The Australian National University, Ngunnawal and Ngambri Country, Canberra, Australia; ^2^Department of Bacteriology, Faculty of Medical Sciences, Tarbiat Modares University, Tehran, Iran

**Keywords:** Academic publishing, ChatGPT, Ethical norms, Mega-journals, Mega-publishers

## Abstract

Those who participate in and contribute to academic publishing are affected by its evolution. Funding bodies, academic institutions, researchers and peer-reviewers, junior scholars, freelance language editors, language-editing services, and journal editors are to enforce and uphold the ethical norms on which academic publishing is founded. Deviating from such norms will challenge and threaten the scholarly reputation, academic careers, and institutional standing; reduce the publishers’ true impacts; squander public funding; and erode the public trust to the academic enterprise. Rigorous review is paramount because peer-review norms guarantee that scientific findings are scrutinized before being publicized. Volunteer peer-reviewers and guest journal editors devote an immense amount of unremunerated time to reviewing papers, voluntarily serving the scientific community, and benefiting the publishers. Some mega-journals are motivated to mass-produce publications and attract the funded projects instead of maintaining the scientific rigor. The rapid development of mega-journals may diminish some traditional journals by outcompeting their impacts. Artificial intelligence (AI) tools/algorithms such as ChatGPT may be misused to contribute to the mass-production of publications which may have not been rigorously revised or peer-reviewed. Maintaining norms that guarantee scientific rigor and academic integrity enable the academic community to overcome the new challenges such as mega-journals and AI tools.

## Introduction

 Scientific communication, reporting, and publishing have been founded on certain ethical norms and standards,^1-3^ which may be affected by many individual or organizational factors. Recognizing and regulating novel factors that challenge the ethical underpinnings of the scientific communication are required for maintaining academic integrity.^4,5^ The ethical dilemmas related to COVID-19 and the pandemic^6-11^ refocused the researchers’ attention to the importance of ethical and transparent sharing of scientific and medical information despite political or regional interests. Presently, two major phenomena—emergence of ChatGPT and perpetuation of mega-journals—jeopardize these ethical norms, consequently affecting the academic or institutional reputations, exhausting the volunteer peer-reviewers or guest editors, compromising the academic integrity and rigorous peer-review, squandering the public funding, and eroding the public trust.

## ChatGPT

 This artificially intelligent language model emerged in November 2022. The potential pros and cons of using ChatGPT or similar AI tools for scientific communication will continually be debated^12,13^ as observed in the scientific literature in 2023. As of 14 June 2023, PubMed of the National Library of Medicine, USA, has listed 599 articles having “ChatGPT” in their title or abstract as opposed to four articles published in 2022, showing the newly heightened academic paranoia and discourse about ChatGPT and similar AI tools. ChatGPT may easily be misused or misappropriated; it can generate flawed or fabricated data, or flawed conclusions that may not befit publication in a scientific journal.^14,15^ Indeed, the ChatGPT developers confirmed that it may produce inaccurate responses causing incorrect conclusions.^16^ Thus, ChatGPT cannot meet the academic authorship standards and requirements. According to many publishers’ guidelines, authors are to declare each co-author’s contributions and their potential conflict of interests. Accordingly, ChatGPT cannot qualify as a“co-author” of academic papers, and a declaration of conflict of interests is a human, not an AI, declaration. ChatGPT challenges the academic publishing enterprise also because of its purported roles in drafting, potentially peer-reviewing (https://www.enago.com/academy/chatgpt-disrupt-peer-review-science-vigilance/), and even editing scientific papers written in English^17^ (https://www.enago.com/academy/negative-costs-of-using-chatgpt-to-edit-research-manuscript/). Without erudite oversight, the very standards of English grammar, syntax, and punctuation expected of the academic communication will degrade in the coming years. Despite some studies on the potential benefits of ChatGPT under educational settings,^18^ use of any AI tool for academic purposes including writing abstracts or academic papers must be with caution, while considering the relevant ethical and societal responsibilities of academic “authorship”.^19,20^ All text generated by ChatGPT must be transparently declared and rigorously double-checked. Nonetheless, ChatGPT may benefit some areas, including writing news articles, tweets, stories for magazines, or poetry.^18^ For example, a book, which contains 105 poems, was written by ChatGPT and is available through Amazon (https://www.amazon.com/105-Poems-ChatGPT-Poetry-Machine-ebook/dp/B0BXHRWR9J). Moreover, an AI tool was reportedly used to enable producing the “final Beatles record” (https://www.bbc.com/news/entertainment-arts-65881813). Although one may not be concerned by reading a poetry book “authored” by ChatGPT or may enjoy listening to an AI-regenerated song by the late John Lennon (Beatles), many scholars will be baffled by seeing a citation to a hypothetical academic publication “co-authored” by ChatGPT discussing some clinical trials of an anti-COVID-19 vaccine. Thus, misuse or unintelligent use of AI-generated technologies jeopardizes the scholarly rigor and integrity, thereby generating mistrust. Establishing and maintaining firm and clear guidelines about the use of ChatGPT or other AI tools for academic writing, scientific communication, or publishing is therefore important.^18-20^

## Mega-journals

 A mega-journal is a peer-reviewed open-access journal that regularly publishes a higher number of papers than a traditional journal yearly.^21^ Other general characteristics of a mega-journal include a wide scope of subjects; a gold model of open-access publishing whereby authors pay an article-processing charge for their manuscript to be published online; publishing a large number of papers in a short period of time; and grounding the peer-review process on scientific soundness instead of the novelty or significance of findings.^22^ Publishing practices by mega-journals and their profit-driven motivations (https://www.theguardian.com/science/2017/jun/27/profitable-business-scientific-publishing-bad-for-science) are the main rationales behind their emergence and development. Now, *Scientific Reports*, *PloS One*, the MDPI *International Journal of Molecular Sciences*, MDPI *Sensors*, MDPI *Molecules*, and *Frontiers in Immunology* are the top six prolific mega-journals^23^ with many publications authored by highly cited researchers. These mega-journals publish thousands of papers yearly, causing concerns about their management and supervision of their peer-review processes. For example, *Oncotarget* published more than 10 000 papers in 2017. After this mega-journal was controversially delisted in 2018 by Clarivate^TM^, its published output was diminished to <200 papers in 2022.^23^ In March 2023, Clarivate^TM^ again delisted dozens of journals published by Hindawi and MDPI when they failed to comply with ethical publishing norms, including publishing outside their scopes, unreasonably fast peer-reviewing, and low rejection rates. For instance, the delisted MDPI journal, *International Journal of Environmental Research and Public Health*, astonishingly published 16 899 papers in 2022.^23^ Although Clarivate^TM^ regularly evaluates indexed journals, delisting such journals surprised and devastated some scholars (https://www.mdpi.com/about/announcements/5536). Such disappointing news sends a warning signal to other scholarly journals with similar publishing practices^23^ and educates also the researchers to reconsider the suitability of a journal before submitting their findings. Thus authors should strongly assess the journals’ impact factors, their editorial practices, and their peer-review grounds before selecting a suitable one for their submission. Although journal impact factor is not the only factor to assess a journal by, and with a focus shifting to, and favoring article-related metrics,^24,25^ assessing and selecting a journal for publishing becomes a challenging task while so many journals and publishers are now available and competing. Thus, this task is better undertaken by senior and experienced academics because less-experienced scholars may choose an inappropriate journal. Utter reliance on journal impact factor may not be wise^26^ or applicable to all disciplines as it can be misused or misinterpreted.^27^

 Reevaluations by Clarivate^TM^ signify the importance of surveilling the scholarly open-access journals that may undermine the peer-review processes or academic integrity. Such reevaluations prohibit an increase in the number of papers published without acceptable ethical standards, threatening the integrity of academic publishing. Surely, markets are influenced by the practices of mega-journals,^23^ and all scholars need training and awareness to choose their intended journals tactfully before they risk being perplexed or affected by another potential delisting.

## Final Remarks

 The ethical norms underpinning the academic publishing standards must be maintained and advocated as publication practices evolve and AI means are developed and deployed. Unethical practices or unethical business opportunities can be realized as a result of essential education about, and advocacy for, ethical publishing norms. Students, scholars, professors, and senior mentors are all well-trained to act ethically when writing a research proposal or undertaking a pilot study. Implementation of ethical practices is even more difficult in the developed or industrialized countries. The numbers of mega-journals, corporations of mega-publishers, and AI tools are increasing and will dominate. The academic environment with the personal desire to achieve highly press the point “to publish or perish.” Unremunerated peer-review will be exhausted and may fail to identify AI-generated manuscripts. Therefore, uniform global guidelines are needed to regulate the future applications or implications of the AI tools within the context of ethical and rigorous academic publishing.

 Can academic rigor and integrity cohabit with AI?
